# Safety and effects of continuous argatroban administration in acute cerebral infarction: a retrospective analysis

**DOI:** 10.1186/s40780-026-00585-z

**Published:** 2026-05-28

**Authors:** Machiko Hirai, Yuki Shigetsura, Daiki Hira, Takakuni Maki, Masayoshi Kawata, Shunsaku Nakagawa, Riki Matsumoto, Tomohiro Terada

**Affiliations:** 1 Department of Clinical Pharmacology and Therapeutics, Kyoto University Hospital, 54 Shogoin-Kawahara-Cho, Sakyo-Ku, Kyoto, 606-8507 Japan; 2 Department of Neurology, Graduate School of Medicine, Kyoto University, 54 Shogoin-Kawahara-Cho, Sakyo-Ku, Kyoto, 606-8507 Japan

**Keywords:** Argatroban, Continuous infusion, Intermittent infusion, Safety, Effectiveness, Stroke

## Abstract

**Background:**

Argatroban is an antithrombotic agent commonly used in the acute phase of cerebral infarction. Although the standard administration method is continuous infusion within the first 2 days only, many patients receive continuous intravenous infusion beyond day 3 owing to concerns of symptoms worsening during intermittent dosing, which may be insufficient given the short half-life of argatroban. However, evidence supporting the continuous intravenous infusion beyond day 3 remains limited. This study aimed to compare the safety and efficacy between continuous and intermittent argatroban administration.

**Methods:**

Patients who received argatroban for acute cerebral infarction at Kyoto University Hospital between January 2019 and August 2021 were retrospectively evaluated. The patients were categorized based on actual treatment regimens into either the intermittent or continuous infusion group. Clinical characteristics, safety outcomes, and neurological status were then compared between the groups. The primary outcome was the incidence of bleeding events, and the secondary outcome was neurological status, as assessed using the manual muscle test.

**Results:**

No clear difference in safety was observed between the two groups. The proportion of patients with a manual muscle test score of 10 significantly increased over time in the intermittent infusion group, whereas no such trend was observed in the continuous infusion group. Subgroup analysis by stroke type showed a similar increasing trend in all groups except the continuous infusion group with branch atheromatous disease, which is known to be associated with a high risk of early neurological deterioration.

**Conclusion:**

Continuous argatroban administration was not associated with an increased risk of bleeding events within the limitations of the current study, despite more intensive concomitant antithrombotic therapy. Although no clear neurological improvement was observed, continuous administration may represent a feasible treatment option in carefully selected patients; however, these findings should be interpreted with caution due to baseline imbalances and potential selection bias, warranting further prospective investigation.

## Background

Acute stroke treatment includes intravenous alteplase administration within 4.5 h of onset [1, 2] and mechanical thrombectomy within 24 h of onset [3]; however, the indications remain limited. Consequently, antiplatelet agents, anticoagulants, cerebroprotective agents, plasma volume expanders, and anti-cerebral edema therapies are frequently used as pharmacological treatments for acute stroke. Among anticoagulants, argatroban is an effective pharmacotherapeutic for acute ischemic stroke owing to its rapid onset, short duration of action, and low bleeding risk [4–9]. Argatroban is an arginine derivative that competitively binds to the active site of thrombin and prevents fibrin deposition. It exerts an immediate anticoagulant effect with a half-life of 30 min, which is rapidly reversed upon drug discontinuation [10].

Argatroban is primarily administered as a continuous intravenous infusion at a dose of 60 mg per day over 24 h during the first 2 days of cerebral infarction treatment, followed by 10 mg twice daily over a 3-h period for 5 days starting from the third day. However, owing to its short half-life, motor paralysis may worsen during the intermittent administration period after day 3 [11]. Consequently, many patients receive continuous intravenous infusion after the third day, with significant symptomatic improvement reported with continuous infusion at a dose of 60 mg/day for 7 days [12, 13]. Despite its clinical use, evidence supporting the off-label use of continuous argatroban infusion remains limited. To date, only one study has directly compared standard administration with continuous infusion beyond day 3 [14]. That study reported no significant differences between the two administration methods with respect to clinical deterioration or bleeding events. However, it did not include neurological assessments, and evidence regarding the impact of different dosing regimens on neurological outcomes remains insufficient.

Thus, this study aimed to compare the safety and effectiveness between continuous and intermittent argatroban administration. Toward this goal, temporal changes in neurological findings and bleeding events during the acute phase of cerebral infarction were analyzed according to the method of argatroban administration.

## Methods

### Study design and ethical considerations

This retrospective study was approved by the Ethics Committee of the Kyoto University Graduate School of Medicine, School of Medicine, and Faculty of Medicine Hospital on September 10, 2021 (approval number: R3165) and was performed in accordance with the Ethical Guidelines for Life Science and Medical Research Involving Human Subjects. Additionally, because the continuous administration of argatroban lacked official approval, permission was granted to neurologists experienced in its application by the Off-Label Drug Evaluation Working Group, established under the Pharmaceutical Safety Management Subcommittee at Kyoto University Hospital. Informed consent was obtained from all patients after providing an explanation of the treatment purpose, potential risks, and benefits.

The decision to initiate continuous argatroban infusion was made based on the clinical judgment of the attending neurologists. These decisions were made as part of routine clinical care and were independent of this study. Treatment selection was based on individual patient conditions, including the risk of neurological deterioration, progression of symptoms, and imaging findings; no standardized criteria were applied. Accordingly, this study was conducted as a retrospective observational analysis of routinely collected clinical data without any intervention assigned for research purposes.

### Patients

Patients admitted to Kyoto University Hospital between January 2019 and August 2021 who experienced cerebral infarction either prior to or during hospitalization were evaluated. Patients who received argatroban for treatment were included. Based on the actual dosing records obtained from electronic medical records, the patients were divided into two groups according to the argatroban administration method: intermittent and continuous infusion. The intermittent infusion group involved patients who received argatroban according to the standard regimen: 60 mg intravenously over 24 h for the first 2 days, followed by 10 mg intravenously over 3 h twice daily for the next 5 days. Meanwhile, the continuous infusion group involved patients who received argatroban 60 mg intravenously over 24 h for the first 3 days, followed by 40 mg intravenously over 16 h on the fourth day. Continuous infusion was not continued beyond day 4, and the total dose of argatroban was equivalent between the continuous and intermittent infusion groups. Patients whose method of administration was not classified under these descriptions were not included in the study.

### Data collection

Data on demographic characteristics and clinical parameters were retrospectively collected from electronic medical records. These included age, sex, body mass index, medical history (stroke, hypertension, diabetes, dyslipidemia, and atrial fibrillation), systolic and diastolic blood pressure, alcohol consumption, and smoking status. Laboratory values at admission such as activated partial thromboplastin time (APTT), prothrombin time-international normalized ratio (PT-INR), and blood glucose levels were also collected. Additionally, information on concomitant medications, including recombinant tissue plasminogen activator, edaravone, glycerin, low-molecular-weight dextran, heparin, anticoagulants, antiplatelets, diuretics, antihypertensives, antidyslipidemia medications, and antidiabetes medications, was obtained. Furthermore, concomitant therapies, such as thrombus retrieval therapy, were also documented.

Considering the potential impact of branch atheromatous disease (BAD) on stroke treatment, the presence or absence of a confirmed or suspected BAD diagnosis was also investigated. Briefly, BAD is an infarction caused by stenosis or occlusion associated with atherosclerotic lesions at the site where a perforating branch diverges from the major artery at the skull base. This condition is associated with progressive cerebral infarction and a poor prognosis [15–17]. In this study, BAD was diagnosed by neurologists based on brain magnetic resonance imaging (MRI) findings during the acute phase of stroke.

### Safety and effectiveness

Safety was evaluated according to the presence or absence of bleeding events during the first 7 days after argatroban administration. Bleeding events were defined as the occurrence of intracerebral hemorrhage, intracerebral microhemorrhage, and gastrointestinal hemorrhage. Enlargement of the infarcted lesion, new infarction, and reocclusion were also evaluated as clinical course outcomes. These events were assessed by a neurologist using MRI findings and medical records. Effectiveness was assessed based on neurological findings during the first 7 days of argatroban administration. Neurological function was evaluated using the Manual Muscle Test (MMT) [18], as this measure was consistently documented in routine clinical records, whereas National Institutes of Health Stroke Scale (NIHSS) scores were not systematically available in this retrospective dataset.

Because both groups received the same argatroban regimen during the first 2 days and the administration schedule diverged thereafter, we focused on temporal changes in neurological status during the treatment period rather than solely on pre–post differences on day 7.

### Statistical analysis

Continuous variables are expressed as median (range) or mean ± standard deviation (SD), depending on their distribution. Categorical variables are presented as counts (percentages). Comparisons between the intermittent and continuous infusion groups were performed using Fisher’s exact test for categorical variables, the Mann-Whitney U test for non-normally distributed continuous variables, and t-test for normally distributed continuous variables. Longitudinal trends in the MMT score distributions were evaluated using the Cochran-Armitage trend test. All statistical analyses were performed using GraphPad Prism 9 (GraphPad Software, La Jolla, CA, USA) or JMP Pro 18 (SAS Institute Inc., Cary, NC, USA). Statistical significance was set at *p* < 0.05.

## Results

### Baseline patient characteristics

The proportion of patients (including those with suspect diagnosis) with BAD was significantly higher in the continuous infusion group than in the intermittent infusion group. Additionally, APTT was longer, and the proportion of individuals with a history of alcohol consumption was significantly higher in the continuous infusion group. Alcohol consumption and smoking status were categorized based on the available medical records, and “unknown” was treated as a separate category in the analysis. These variables were included for descriptive purposes, and their interpretation was limited due to missing information. Table 1 presents the patient characteristics at the start of argatroban administration.

**Table 1:** Comparison of baseline patient characteristics between the intermittent and continuous infusion groups

	Intermittent infusion group *n* = 90	Continuous infusion group *n* = 50	*p*-value
Sex (male/female), *n*	62/28	39/11	0.33^a^
Age (years), median (range)	73.0 (67.0–82.0)	71.5 (67.0–82.3)	0.92^b^
BMI (kg/m^2^), median (range)	23.1 (20.7–25.1)	21.8 (20.6–27.0)	0.95^b^
History of cerebral infarction (first occurrence/recurrence), n	65/25	36/14	1.0^a^
BAD (including suspect cases), n (%)	40 (44.4)	34 (68.0)	0.008^a^
Laboratory values			
APTT, mean ± SD	27.8 ± 3.7	29.4 ± 4.3	0.014^b^
PT-INR, mean ± SD	1.0 ± 0.1	1.0 ± 0.1	0.24^b^
Systolic blood pressure (mmHg), mean ± SD	165.8 ± 28.7	160.7 ± 26.2	0.50^c^
Diastolic blood pressure (mmHg), mean ± SD	91.9 ± 19.6	90.26 ± 18.4	0.63^c^
Blood glucose level (mg/dL), mean ± SD	149.0 ± 64.5	149.1 ± 75.9	0.62^b^
Comorbidities, n (%)			
Hypertension	67 (74.4)	40 (80.0)	0.54^a^
Diabetes mellitus	39 (43.3)	17 (34.0)	0.37^a^
Dyslipidemia	54 (60.0)	31 (62.0)	0.86^a^
Atrial fibrillation	10 (11.1)	4 (8.0)	0.57^a^
Alcohol consumption (none /occasional/habitual/unknown), n	33/11/38/8	13/16/20/1	0.021^a^
Smoking status (none/past/current/unknown), n	35/30/22/3	13/19/18/0	0.21^a^

^a^Fisher’s exact test, ^b^Mann–Whitney U test, ^c^*t*-test

BMI, body mass index; BAD, branch atheromatous disease; APTT, activated partial thromboplastin time; PT-INR, prothrombin time-international normalized ratio

### Medications and concomitant therapy after argatroban initiation

Table 2 shows the medications administered after the initiation of argatroban administration. The proportion of patients who received concomitant antiplatelet agent treatment was significantly higher in the continuous infusion group than in the intermittent infusion group, with 35 (70%) patients and 12 patients (24%) in the continuous infusion group receiving dual antiplatelet therapy (DAPT) and triple antiplatelet therapy (TAPT), respectively. With respect to the antiplatelet agents used, the frequency of clopidogrel and aspirin use did not differ between the two groups, whereas cilostazol, dextran, and heparin were prescribed more frequently in the continuous infusion group. In addition, calcium channel antagonists and statins were used significantly more frequently in the intermittent infusion group.

**Table 2:** Comparison of medications administered after the initiation of argatroban administration between the intermittent and continuous infusion groups

Medications, *n* (%)		Intermittent infusion group *n* = 90	Continuous infusion group *n* = 50	*p*-value
Antiplatelet agents	Clopidogrel	71 (78.9)	41 (82.0)	0.83
	Aspirin	84 (93.3)	49 (98.0)	0.42
	Cilostazol	16 (17.8)	22 (44.0)	0.001
	None SAPT DAPT TAPT	1 (1.1) 10 (11.1) 79 (87.8) 0 (0)	0 (0) 3 (6.0) 35 (70.0) 12 (24.0)	< 0.0001
Anticoagulants	Rivaroxaban	0 (0)	2 (4.0)	0.13
	Edoxaban	1 (1.1)	2 (4.0)	0.23
	Apixaban	1 (1.1)	0 (0)	1.0
Thrombectomy therapy		4 (4.4)	0 (0)	0.30
rt-PA		6 (6.7)	3 (6.0)	1.0
Edaravone		59 (65.6)	38 (76.0)	0.25
Glycerin		1 (1.1)	0 (0)	1.0
Dextran		5 (5.6)	12 (24.0)	0.0023
Heparin		4 (4.4)	25 (50.0)	< 0.0001
Diuretics		6 (6.7)	2 (4.0)	0.71
Antihypertensive drugs	Calcium channel blockers	27 (30.0)	5 (10.0)	0.007
	ACE inhibitors	2 (2.2)	0 (0)	0.54
	ARB	16 (17.8)	4 (8.0)	0.14
	β blockers	7 (7.9)	3 (6.0)	1.0
Antidyslipidemia medications	Statins	66 (73.3)	45 (50.0)	0.002
	Fibrates	1 (1.1)	0 (0)	1.0
Antidiabetes medications		27 (30.0)	11 (22.0)	0.33

Fisher’s exact test

SAPT, single antiplatelet therapy; DAPT, dual antiplatelet therapy; TAPT, triple antiplatelet therapy; rt-PA, recombinant tissue plasminogen activator; ACE, angiotensin-converting enzyme inhibitor; ARB, angiotensin II receptor blocker

### Safety and clinical course outcomes

Table 3 presents the frequency of bleeding events and the occurrence of infarct enlargement, new infarcts, and reocclusion following the initiation of argatroban treatment. Only a small number of patients experienced bleeding events in both groups, and the difference was not significant. Additionally, the infarct size, occurrence of new infarcts, and reocclusion were not significantly different between the two groups.

**Table 3:** Comparison of safety and clinical course outcomes between the intermittent and continuous infusion groups

	Intermittent infusion group *n* = 90	Continuous infusion group *n* = 50	*p*-value
Safety outcomes			
Hemorrhagic complications	3 (3.3)	3 (6.0)	0.67
Asymptomatic microbleeds	1	0	
Intrainfarct hemorrhage	2	3	
Gastrointestinal bleeding	0	0	
Clinical course outcomes			
Infarct expansion, new infarction, or reocclusion	18 (20.0)	16 (32.0)	0.15

Fisher’s exact test

### Changes in neurological findings

**Fig. 1:**
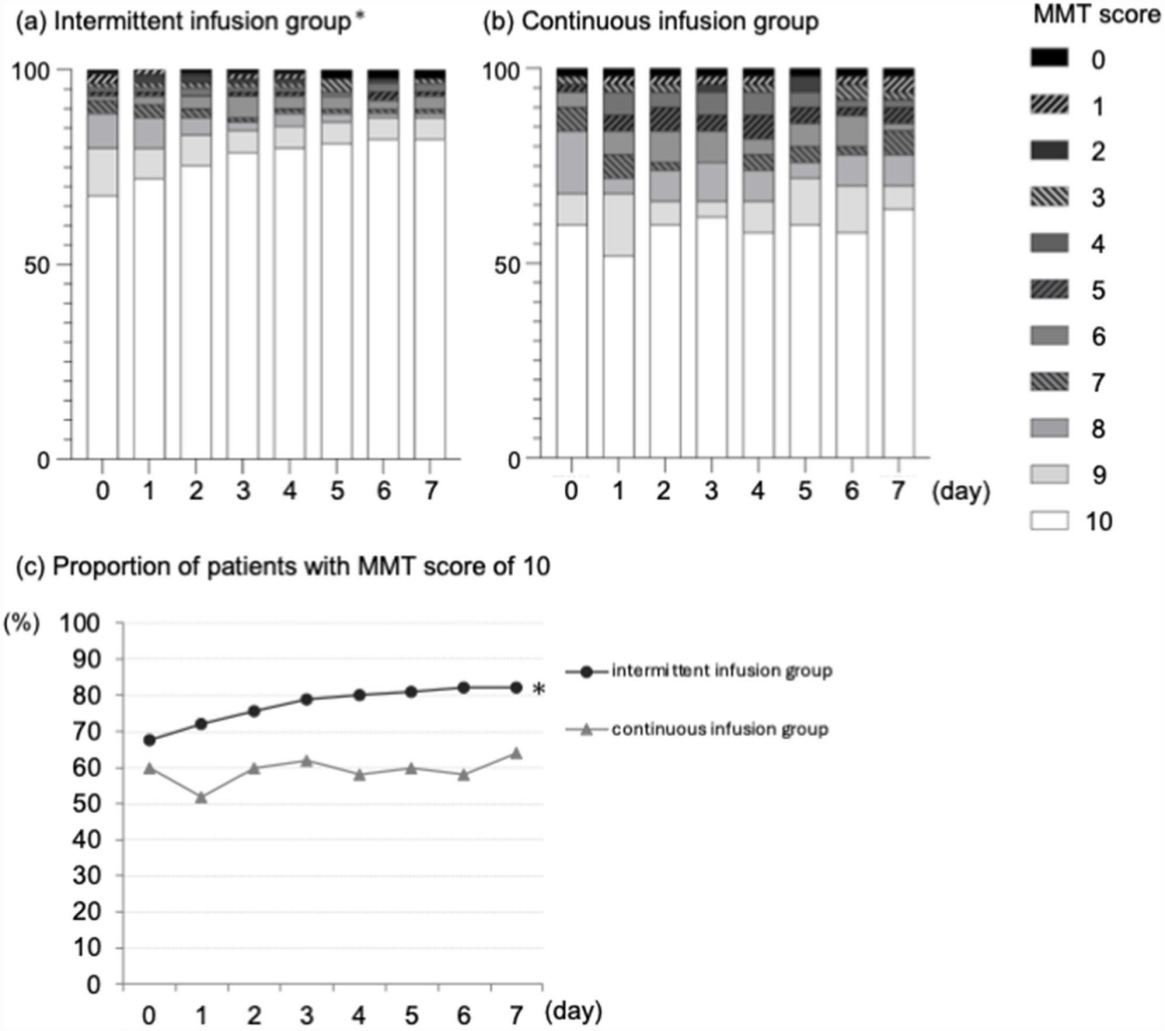
Manual Muscle Test (MMT) scores by administration method. The figure illustrates the distribution of MMT scores over the first 7 days of argatroban administration, stratified by administration method. (**a**) Intermittent infusion group (*n* = 90). (**b**) Continuous infusion group (*n* = 50). (**c**) Line graph highlighting the proportion of patients with an MMT score of 10 in each group over time. The Cochran-Armitage trend test shows a significant upward trend in the intermittent infusion group (*p* < 0.05)

**Fig. 2:**
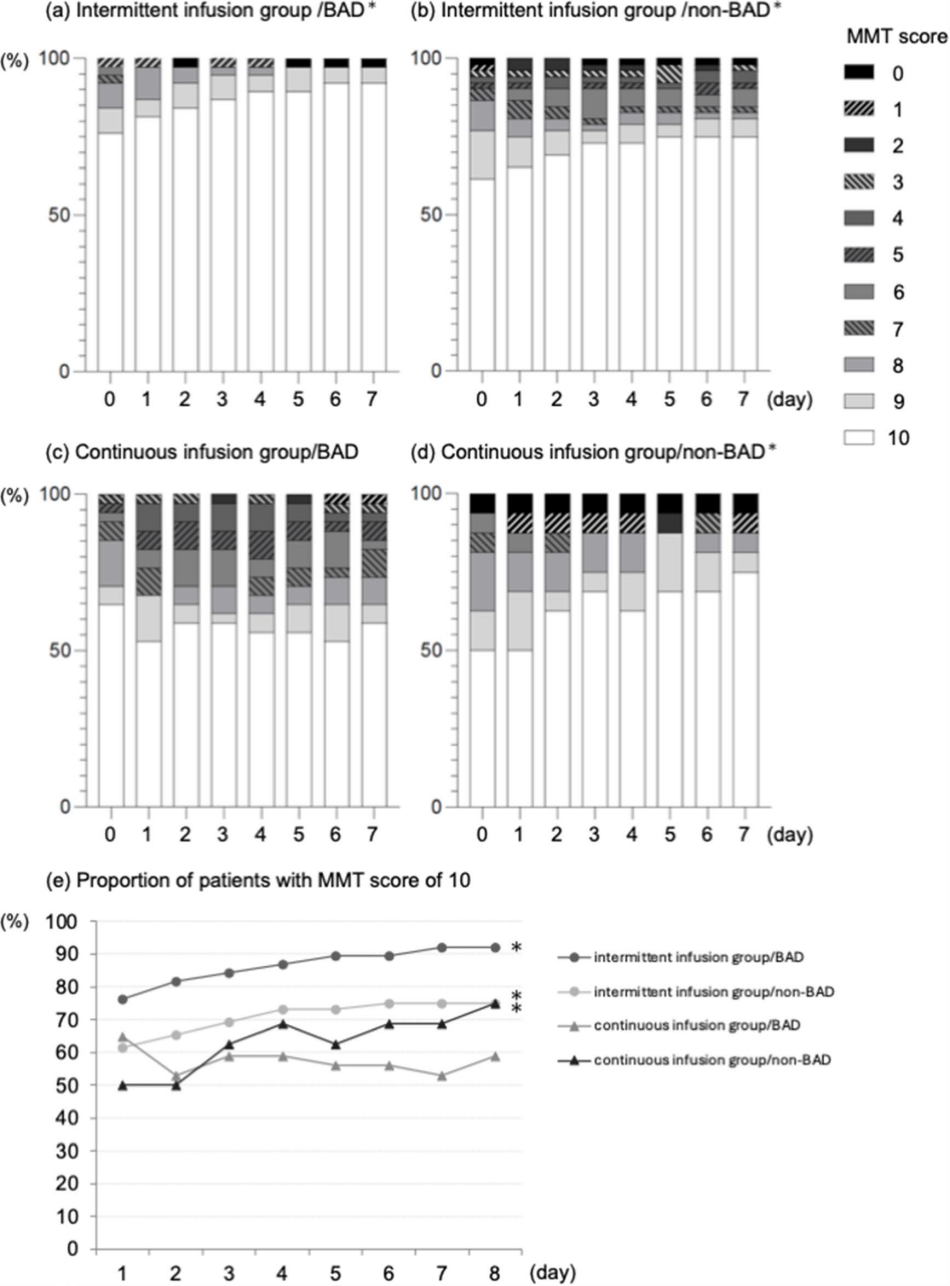
Manual Muscle Test (MMT) scores by stroke type subgroup. The figure illustrates the distribution of MMT scores over the first 7 days of argatroban administration in the intermittent and continuous infusion groups stratified by stroke type. (**a**) Intermittent infusion group/ branch atheromatous disease (BAD) (*n* = 40). (**b**) Intermittent infusion/non-BAD (*n* = 50). (**c**) Continuous infusion group/BAD (*n* = 34). (**d**) Continuous infusion group/non-BAD (*n* = 16). (**e**) Line graph highlighting the proportion of patients with MMT score of 10 in each group. The Cochran-Armitage trend test shows a significant difference in the trends among the subgroups (intermittent infusion/BAD subgroup vs. intermittent infusion/non-BAD subgroup vs. continuous infusion/non-BAD subgroup, *p < 0.05), except for the continuous infusion group/BAD subgroup

MMT scores were obtained for all included patients, and no missing data were noted for this variable. Figure 1 shows the trends in MMT scores for both the intermittent and continuous infusion groups during the first 7 days after treatment initiation. An MMT score of 10 in this study was interpreted as indicating full motor function in the affected limb. A significant upward trend in the proportion of patients with an MMT score of 10 was observed in the intermittent infusion group (*p* < 0.05), whereas no such trend was found in the continuous infusion group, as determined by the Cochran-Armitage trend test. Given the background differences between patients, particularly in stroke subtype, a similar analysis was performed after stratifying patients by stroke type (BAD or non-BAD), as shown in Fig. 2. The results indicated a significant upward trend (*p* < 0.05) in the proportion of patients who achieved an MMT score of 10 in the intermittent infusion/BAD subgroup, intermittent infusion/non-BAD subgroup, and continuous infusion/non-BAD subgroup. However, this trend was not observed in the continuous infusion/BAD subgroup (Fig. 2C).

## Discussion

Argatroban is a commonly used antithrombotic agent in the acute phase of cerebral infarction [4–9]; however, it is frequently administered in a manner different from standard indications. Despite its widespread off-label use, evidence supporting alternative administration methods remains limited, and treatment decisions have been largely based on the physician’s discretion and case reports [12, 13]. In this study, no clear difference in safety was observed between continuous and intermittent argatroban infusion. In contrast to previous studies [14], which evaluated neurological deterioration as a binary outcome, our study included a temporal assessment of neurological function using MMT over the first 7 days of treatment. This approach allowed us to capture changes in motor function over time and provided additional insight into the clinical course of neurological recovery.

However, differences in baseline patient characteristics between the groups, particularly the higher proportion of patients with BAD and more intensive concomitant therapies in the continuous infusion group, may reflect treatment selection for patients at a higher risk of neurological deterioration. In addition, the use of intensive concomitant antithrombotic therapies may have contributed to the suppression of neurological deterioration and may have masked any potential effect of the continuous infusion itself, making it difficult to isolate its specific contribution. Nevertheless, in real-world clinical practice, treatment strategies are determined based on the overall clinical condition of the patient, and the present findings suggest that, under such conditions, continuous infusion does not result in apparent neurological worsening; however, these findings should be interpreted with caution.

In the continuous group, the first 2 days of administration followed the same protocol as that in the intermittent infusion group; however, the doses on days 3 and 4 were higher than those in the intermittent infusion group. Consequently, the primary concern with the continuous infusion regimen is the increased risk of hemorrhage. Patients are often treated with antiplatelet agents during the acute phase of cerebral infarction. DAPT is recommended as short-term treatment for patients with acute ischemic stroke or transient ischemic attack (TIA) [19]. Additionally, a combination of three antiplatelet agents (aspirin, clopidogrel, and dipyridamole) within 2 days of stroke onset may enhance antiplatelet effectiveness in patients with noncardiogenic stroke or TIA. However, this regimen does not prevent stroke or recurrent TIA; it is also associated with a significantly higher risk of bleeding than clopidogrel alone or aspirin plus dipyridamole [20].

In the current study, TAPT was administered only in the continuous infusion group, and none of the patients in the intermittent infusion group received TAPT. In addition, more patients in the continuous infusion group were administered heparin. In a randomized controlled trial of 418 patients with non-lacunar stroke, the proportion of patients achieving a modified Rankin Scale score of ≤ 2 at 3 months was significantly higher in those who received unfractionated heparin than in those who received saline infusion within 3 h of stroke onset [21]. However, this benefit was also accompanied by a significant increase in symptomatic intracranial hemorrhage. These findings indicate a possibly higher risk of bleeding in the continuous infusion group than in the intermittent infusion group in the current study. However, no apparent increase in bleeding events was observed in the continuous infusion group within the limitations of the current study scale.

A trend analysis of the proportion of patients with an MMT score of 10 showed a significant increase over time in the intermittent infusion group, whereas no such trend was observed in the continuous infusion group. However, differences in patient backgrounds and treatment profiles were observed between the groups, particularly with respect to distribution of BAD, which may have influenced these findings and should be considered when interpreting the apparent lack of neurological improvement. BAD was first proposed by Caplan [22] in 1989 as the cause of perforator territory infarctions. BAD is an infarction resulting from stenosis or occlusion due to atherosclerotic lesions at the site where the perforating artery branches off from the main trunk artery at the base of the skull. A study comparing patients with BAD and those with lacunar infarction found a higher rate of disease progression in patients with BAD than in those with lacunar infarction (51% vs. 22%), indicating a significantly higher risk of progression in patients with BAD [23]. A comparative study of small deep brain infarcts classified into BAD and non-BAD types reported significantly higher rates of progressive stroke in BAD-type patients (36.7% vs. 12.5%) [24]. These findings indicate that BAD is associated with progressive cerebral infarction and poor acute outcomes [15]. In the present study, continuous treatment did not demonstrate clear neurological improvement in BAD, whereas improvement was observed in the intermittent infusion group. However, this difference should be interpreted with caution, as patients with BAD in the continuous infusion group may have had more severe or progressive disease and were more likely to receive intensive concomitant therapies, reflecting treatment selection based on clinical judgment in routine practice. These factors may have influenced the observed outcomes, making it difficult to determine whether the differences reflected the effect of the administration method itself. Therefore, the effect of continuous administration in BAD should be interpreted with caution.

This study has limitations. First, the single-center design may have introduced a selection bias in comparisons between continuous and intermittent dosing. In particular, patients with more severe or progressive disease may have been preferentially treated with continuous infusion, which could have influenced both the treatment selection and outcomes. In addition, differences in concomitant therapies further limit the ability to attribute the observed effects solely to the administration method. Additionally, the sample size was small, limiting the ability to adequately assess rare adverse events, such as bleeding. Second, stroke severity was not objectively assessed. This study did not use the NIHSS, a standard measure of stroke severity. Instead, the analysis was based solely on neurological status assessed using the MMT score, which may not fully capture stroke severity, as it primarily reflects motor function and does not capture non-motor deficits, such as sensory impairment or aphasia. Third, this is a retrospective study. In clinical practice, treatment is tailored for individual patient symptoms, and the most appropriate therapy is selected accordingly. Therefore, it was impossible to isolate the effects of argatroban. A randomized controlled trial is needed to more accurately evaluate the efficacy and safety of continuous versus intermittent argatroban administration.

## Conclusions

The present study did not demonstrate superiority of continuous infusion in terms of neurological recovery. However, our findings suggest that no apparent increase in bleeding risk was observed even in patients with BAD who were frequently treated with multiple antithrombotic agents within the limitations of the current study scale. These findings should be interpreted with caution, as baseline imbalances and potential selection bias may have influenced the outcomes. Larger prospective studies are required to confirm these findings and determine the optimal dosing strategy.

## Data Availability

The datasets analyzed during the current study are not publicly available due to their containing information that could compromise the privacy of patients but are available from the corresponding author on reasonable request.

## Abbreviations

APTT: Activated partial thromboplastin time
BAD: Branch atheromatous disease
DAPT: Dual antiplatelet therapy
MMT: Manual muscle test
MRI: Magnetic resonance imaging
NIHSS: National Institutes of Health Stroke Scale
PT-INR: Prothrombin time-international normalized ratio
TAPT: Triple antiplatelet therapy
TIA: Transient ischemic attack
